# Axl is required for TGF-β2-induced dormancy of prostate cancer cells in the bone marrow

**DOI:** 10.1038/srep36520

**Published:** 2016-11-07

**Authors:** Kenji Yumoto, Matthew R. Eber, Jingcheng Wang, Frank C. Cackowski, Ann M. Decker, Eunsohl Lee, Ana Rita Nobre, Julio A. Aguirre-Ghiso, Younghun Jung, Russell S. Taichman

**Affiliations:** 1 Department of Periodontics and Oral Medicine, University of Michigan School of Dentistry, Ann Arbor, MI 48109, USA; 2 Department of Cancer Biology and Comprehensive Cancer, Wake Forest University School of Medicine, Winston-Salem, NC, USA; 3 Department of Internal Medicine, Division of Hematology and Oncology, University of Michigan School of Medicine, Ann Arbor, MI 48109, USA; 4 Departments of Medicine, Division of Hematology and Oncology, Otolaryngology and Oncological Sciences, Head and Neck Cancer Basic Research, Departments of Otolaryngology, NCI-Designated Tisch Cancer Institute at Mount Sinai, Icahn School of Medicine at Mount Sinai New York, NY10029, USA.

## Abstract

Disseminated prostate cancer (PCa) cells in the marrow survive for years without evidence of proliferation, while maintaining the capacity to develop into metastatic lesions. These dormant disseminated tumor cells (DTCs) may reside in close proximity to osteoblasts, while expressing high levels of Axl, one of the tyrosine kinase receptors for growth arrest specific 6 (Gas6). Yet how Axl regulates DTC proliferation in marrow remains undefined. Here, we explored the impact of the loss of Axl in PCa cells (PC3 and DU145) on the induction of their dormancy when they are co-cultured with a pre-osteoblastic cell line, MC3T3-E1. MC3T3-E1 cells dramatically decrease the proliferation of PCa cells, however this suppressive effect of osteoblasts is significantly reduced by the reduction of Axl expression in PCa cells. Interestingly, expression of both TGF-β and its receptors were regulated by Axl expression in PCa cells, while specific blockade of TGF-β signaling limited the ability of the osteoblasts to induce dormancy of PCa cells. Finally, we found that both Gas6 and Axl are required for TGF-β2-mediated cell growth suppression. Taken together, these data suggest that a loop between the Gas6/Axl axis and TGF-β2 signaling plays a significant role in the induction of PCa cell dormancy.

Bone marrow (BM) metastases are a major cause of death in prostate cancer (PCa) patients^1^,^2^. They are largely the result of the reactivation of disseminated tumor cells (DTCs) which escape early in the disease progression, yet have remained dormant for years^3^. It is a significant problem that DTCs in BM often become resistant to current cancer chemotherapies which target actively proliferating cancer cells. In order to prevent PCa metastases in the BM, it is therefore important to understand how DTCs become dormant and under what conditions they escape the proliferative regulation imposed by the marrow. Although recent studies have defined some of key molecules and signaling pathways which regulate tumor cell dormancy^4^,^5^, much remains to be understood.

Recently, two members of the TGF-β superfamily (TGF-β2 and BMP-7) were shown to regulate DTC dormancy in the BM^6^,^7^ and BMP-4 also regulates dormancy in the lung^8^. TGF-β2 induces the expression of p27, a potent endogenous cell cycle inhibitor, by increasing phosphorylation of p38 and activation of Smad2 and Smad1/5^6^. Yet, even TGF-β signaling has divergent roles in regulating tumor proliferation and most of the studies have focused on TGF-β1 isoform when elucidating these mechanisms. For example, TGF-β1 in early stage lesions suppresses cellular proliferation, while it promotes tumor progression in late stages^9^,^10^,^11^,^12^,^13^. Recently it was shown that TGF-β2 was upregulated in the DTCs isolated from PCa patients that had no evidence of disease from 7–18 years after radical prostatectomy, compared to DTCs from patients with active PCa metastatic disease^14^. TGF-β2, while less understood compared to TGF-β1, seems to induce growth suppression even in cancer cells with highly altered genomes^6^,^14^, suggesting that isoform function might shed light into how some microenvironments can still impose dormancy of aggressive cancer cells. Similarly, BMP-7 is produced by bone marrow stromal cells and it can suppress the proliferation of PCa cells by activating p38 and increasing the cell cycle inhibitor expression, including p21 and p27^7^. The suppressive effect on cell proliferation by BMP-7 depends on the BMP receptor 2 (BMPR2), and BMPR2 expression inversely correlates with bone metastases in prostate cancer patients^7^.

In prior studies, we reported that the process of metastasis is similar to the homing behavior of hematopoietic stem cells (HSCs) in the marrow where DTCs target and engage the HSC niche during dissemination^15^. Like HSCs, once DTCs are engaged in the osteoblastic niche, they become quiescent. Taking further clues from HSCs-niche interactions, we have found that like HSCs, growth arrest specific 6 (Gas6), regulates DTC quiescence and tumor development^16^,^17^. Gas6 signaling is dependent on at least three tyrosine kinase receptors including Tyro3, Axl and MerTK (TAM receptors)^18^,^19^. Using a xenograft model of tumor dormancy we found that high expression of Axl relative to Tyro3 is associated with PCa cell dormancy in the marrow, suggesting that Axl signaling may be associated with the induction of a dormant phenotype^20^.

In the present study we further explored the role that Axl plays in PCa cellular dormancy, using a co-culture system in which PCa cells become quiescent on the pre-osteoblastic cell line, MC3T3-E1. We found that MC3T3-E1 cells dramatically enhance the dormancy signature of PCa cells recovered from the co-culture, however, loss of Axl expression in PCa cells limits the induction of the dormant phenotype. Interestingly, loss of Axl expression also leads to decreased expression of TGF-β ligands and TGF-β receptor 2 (TGFBR2) by PCa cells in the co-culture, while TGF-β signaling limits PCa proliferation in the co-culture system. More specifically, we found that TGF-β2, but not TGF-β1 could induce Gas6 and p27 expression in PC3 cells. Furthermore, Gas6 and Axl expression are required for TGF-β2-mediated PCa cell growth suppression. Our results strongly suggest that there is a critical crosstalk between TGF-β and Gas6/Axl signaling pathways to regulate PCa cell dormancy.

## Results

### MC3T3-E1 pre-osteoblasts induce PCa cell dormancy

**Figure 1:**
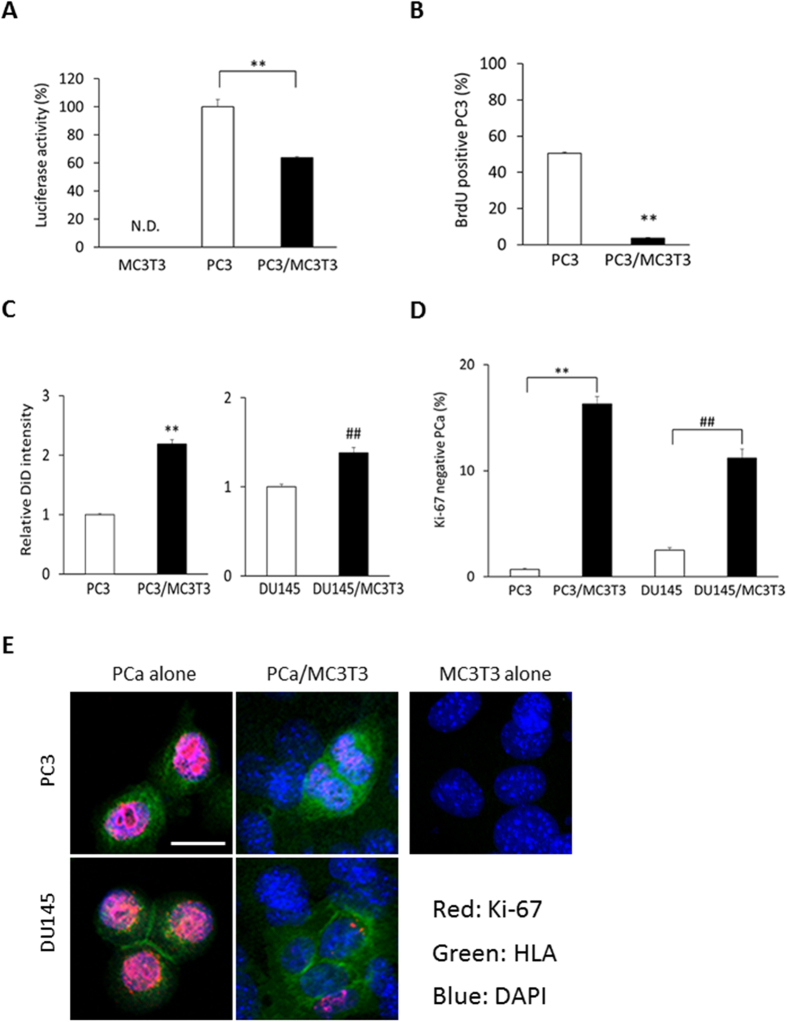
MC3T3-E1 cells induce a dormancy signature in PCa cells. (**A**) Luciferase activity in PC3 cells 4 days after co-culture with MC3T3-E1 cells. **p < 0.01, compared to PC3 cells cultured alone, N.D. means not detected. (**B**) BrdU incorporation in PC3 cells 4 days after co-culture with MC3T3-E1 cells by flow cytometry. **p < 0.01, compared to PC3 cells cultured alone. (**C**) Both PC3 and DU145 cells were pre-labeled with a fluorescent dye, DiD, and co-cultured with MC3T3-E1 cells. DiD levels in PCa cells were measured by FACS 6 days after the co-culture was initiated. **p < 0.01 compared to PC3 cells cultured alone, ^##^p < 0.01 compared to DU145 cells cultured alone. (**D**) Ki-67 negative populations in PCa cells 4 days after the co-culture evaluated by flow cytometry. **p < 0.01 compared to PC3 cells cultured alone, ^##^p < 0.01 compared to DU145 cells cultured alone. (**E**) Immunocytochemistry showing Ki-67 expression. PCa cells were recognized by HLA with green color, and Ki-67 was shown with red color. Scale bar, 20 μm.

Osteoblasts are critical components of the HSC niche and are known to play a significant role in regulating disseminated tumor cell (DTC) proliferation in the marrow^21^. To study the mechanisms underlying the induction of dormancy by osteoblasts, we tested whether osteoblasts can induce a dormant state in PCa cells *in vitro*. Luciferase expressing PCa cells were cultured on subconfluent MC3T3-E1 cells, and PCa cell proliferation was evaluated. After four days of co-culture, luciferase activity was significantly lower in the PC3 co-cultured with MC3T3-E1 cells relative to PC3 cells cultured alone (Fig. 1A). To confirm that a dormant phenotype was established in PCa cells by the co-culture, flow cytometry was used to analyze BrdU incorporation. For these studies, PCa cells were distinguished from the murine MC3T3-E1 cells by antibodies against the human leukocyte antigen (HLA). BrdU incorporation was dramatically decreased in PC3 cells co-cultured with osteoblasts compared to PC3 cells alone (Fig. 1B). Next, PCa cells were labeled with DiD to evaluate proliferation in live cells. DiD is a fluorescent dye, which binds to cell membranes and is equally distributed into daughter cells during proliferation while high label retention represents cells which have undergone fewer divisions^22^. More label was retained by the PC3 and DU145 cells when they were co-cultured with MC3T3-E1, compared to those cells cultured alone (Fig. 1C). Furthermore, Ki-67 expression was evaluated as it is expressed in G1, S, G2 and M stages of the cell cycle, but not in the G0, dormant state. Ki-67 negative populations were significantly increased when the PCa cells were co-cultured with osteoblasts (Fig. 1D). Consistent with the FACS data, Ki-67 expression levels were significantly suppressed in PCa cells after 3 days of the co-culture when examined them by immunocytochemistry (Fig. 1E). Together, these data indicate that MC3T3-E1 cells suppressed the proliferation of the PCa cells.

### TAM receptor expression in the slow-cycling PCa cells

**Figure 2:**
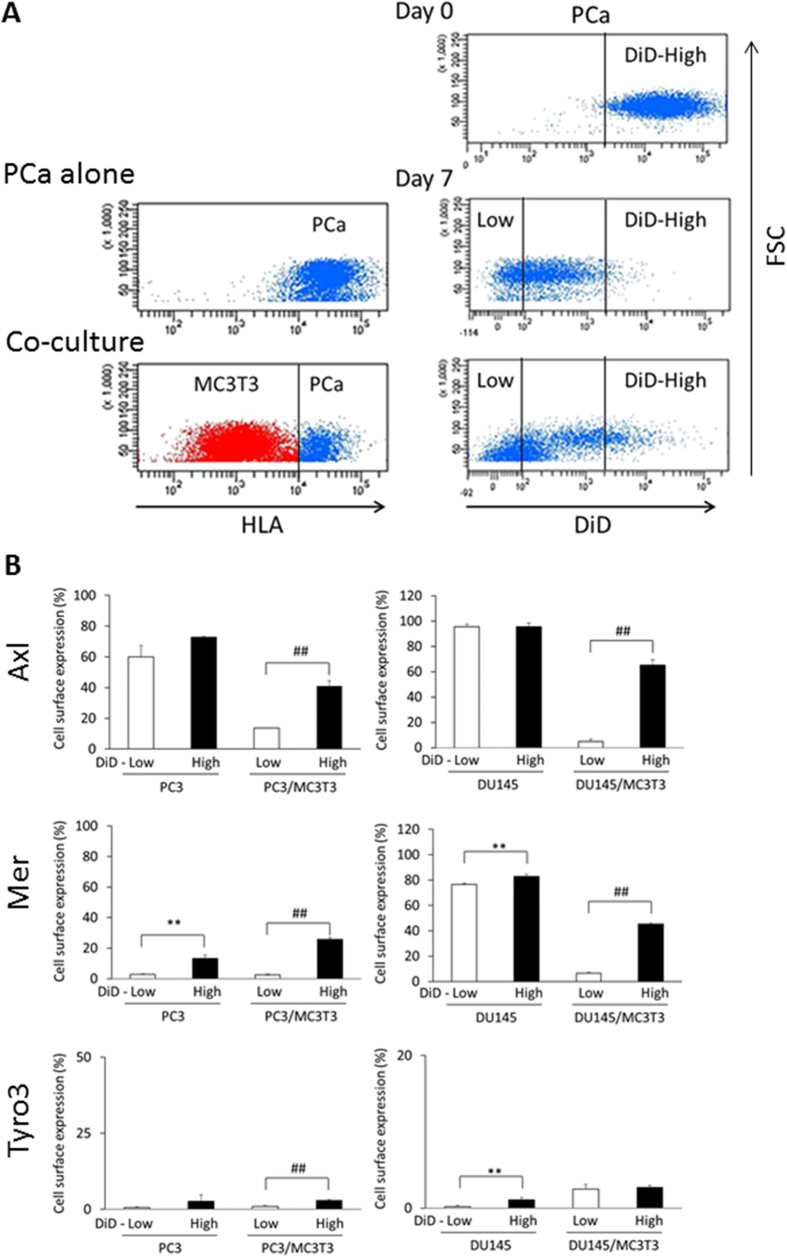
TAM receptor expression in the slow-cycling PCa cells. (**A**) Selection of PCa cells as HLA-positive populations and DiD fluorescence levels in PCa cells by flow cytometry in co-culture with MC3T3-E1 cells. DiD fluorescence levels were examined in PCa cells before and 7 days after the co-culture. (**B**) Cell surface expression of TAM receptors between DiD-low and DiD-high PCa cells cultured in the presence or absence of MC3T3-E1 cells. **p < 0.01 compared to DiD-low PCa cells cultured in the absence of MC3T3-E1 cells, ^##^p < 0.01 compared to DiD-low PCa cells cultured in the presence of MC3T3-E1 cells.

Gas6, the ligand of the TAM receptors, regulates DTC quiescence and tumor development in PCa^16^,^17^. Gas6 is abundantly secreted from MC3T3-E1 cells, but not in PC3 or DU145 cells, although it is detected in the cell lysates and on the cell surface of those PCa cells (Fig. S1). Therefore, Gas6 secreted from MC3T3-E1 cells may suppress the proliferation of PCa cells through TAM receptors expressed by PCa cells in our co-culture systems. Axl expression is associated with a non-proliferative state of DTCs in the bone marrow^20^. To test whether Axl expression levels are correlated with cellular dormancy in our co-culture model system, cell surface expression of the TAM receptors was examined by flow cytometry. For these studies, PCa cells were pre-labeled with DiD, and then cultured with MC3T3-E1. After 7 days, HLA positive PCa cells were selected and DiD retention levels were detected in PCa cells (Fig. 2A). Each of the TAM receptors expression was examined between DiD-low (fast-cycling) and DiD-high (slow-cycling) PCa cells (Fig. 2B). Significantly higher expression of Axl is detected in slow-cycling cells compared to fast-cycling cells both in PC3 and DU145 when they are cultured in the presence of osteoblasts, although there is no difference in Axl expression between fast and slow-cycling PCa cells when they are cultured in the absence of osteoblasts (Fig. 2B). Yet Mer expression is higher in the slow-cycling PCa cells both in the presence and absence of osteoblasts, and Tyro3 levels are slightly higher in slow-cycling cells in PC3, but not in DU145 in the presence of osteoblasts (Fig. 2B). The higher ratio Axl/Tyro was correlated with slow-cycling cells in DU145, but not in PC3 (Fig. S2), while a previous study by our group demonstrated that the higher ratio of Axl/Tyro3 is associated with the dormant DTCs extracted from bone marrow^20^.

### Axl knockdown reduced dormant cell induction in PCa cells during the co-culture

**Figure 3:**
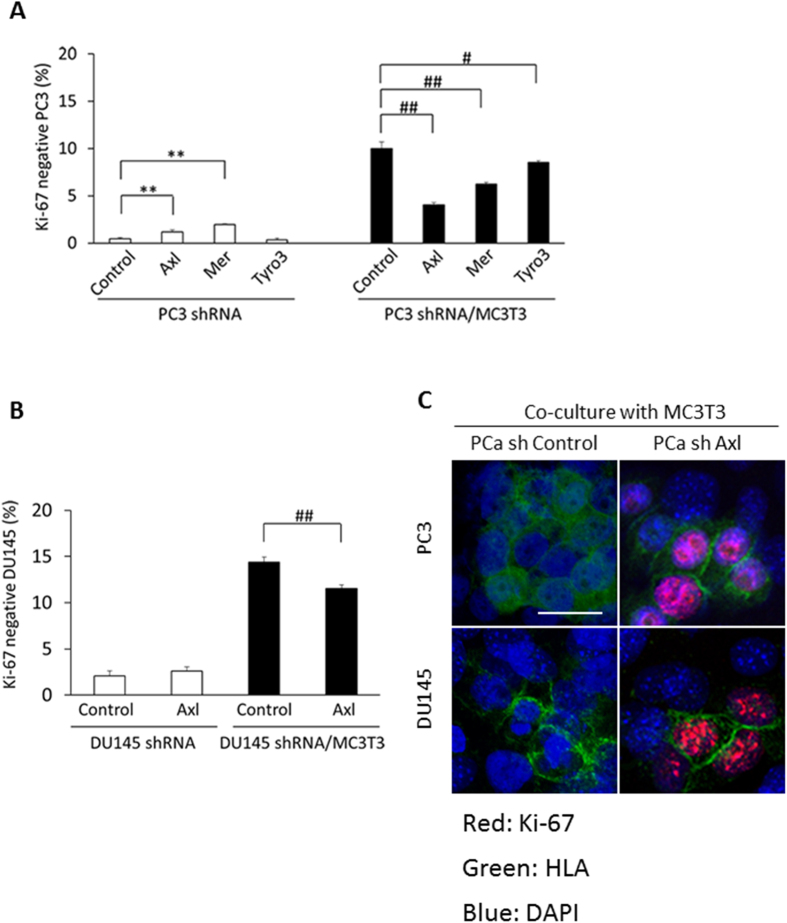
Ki-67 expression levels in each of TAM receptor knockdown PCa cells 4 days after co-culture with MC3T3-E1 cells. (**A**) Ki-67 negative populations in PC3 cells after the co-culture. **p < 0.01 compared to PC3 sh control cells cultured alone, ^#^p < 0.05, ^##^p < 0.01 compared to PC3 sh control cells co-cultured with MC3T3-E1 cells. (**B**) Ki-67 negative population in DU145 cells after the co-culture. ^##^p < 0.01 compared to DU145 sh control cells co-cultured with MC3T3-E1 cells. (**C**) Immunocytochemistry showing Ki-67 expression. PCa cells were recognized by HLA with green color, and Ki-67 was shown with red color. Scale bar, 20 μm.

PCa cells become slow-cycling or dormant when they are cultured with MC3T3-E1cells, while TAM receptor expression is enhanced in the dormant or slow-cycling PCa cells. To examine whether TAM receptors are involved in the induction of PCa dormancy in the co-culture, Tyro3, Axl and Mer were knocked down using shRNA technology (sh Tyro3, sh Axl and sh Mer) and the cells were co-cultured with MC3T3-E1. Reduction of each TAM receptor expression was confirmed by western blots (Fig. S3). Four days later, Ki-67 negative PC3 cells were evaluated. Although Ki-67 negative populations were dramatically increased in PC3 sh Control cells cultured on MC3T3-E1 cells, fewer Ki-67 negative cells were detected when Tyro3, Axl and Mer levels were reduced by sh RNA (Fig. 3A). Importantly, reduction in Axl expression had the predominant impact on Ki-67 negative populations, suggesting that Axl plays a pivotal role in dormancy induction (Fig. 3A). Likewise, Axl knock down in DU145 cells partially suppressed Ki-67 negative populations during the co-culture (Fig. 3B). Next, PCa cells were stained with HLA-specific antibodies, and Ki-67 expression was detected using anti-Ki-67 antibodies by immunohistochemistry. Knockdown of Axl in both PC3 and DU145 resulted in significantly higher levels of Ki-67 staining compared to control cells in the co-cultures (Fig. 3C). Based on these results, we focused further attention on the role of Axl in tumor cell dormancy.

### Enhanced expression of TGF-β ligands and receptors by PCa cells during co-culture

**Figure 4:**
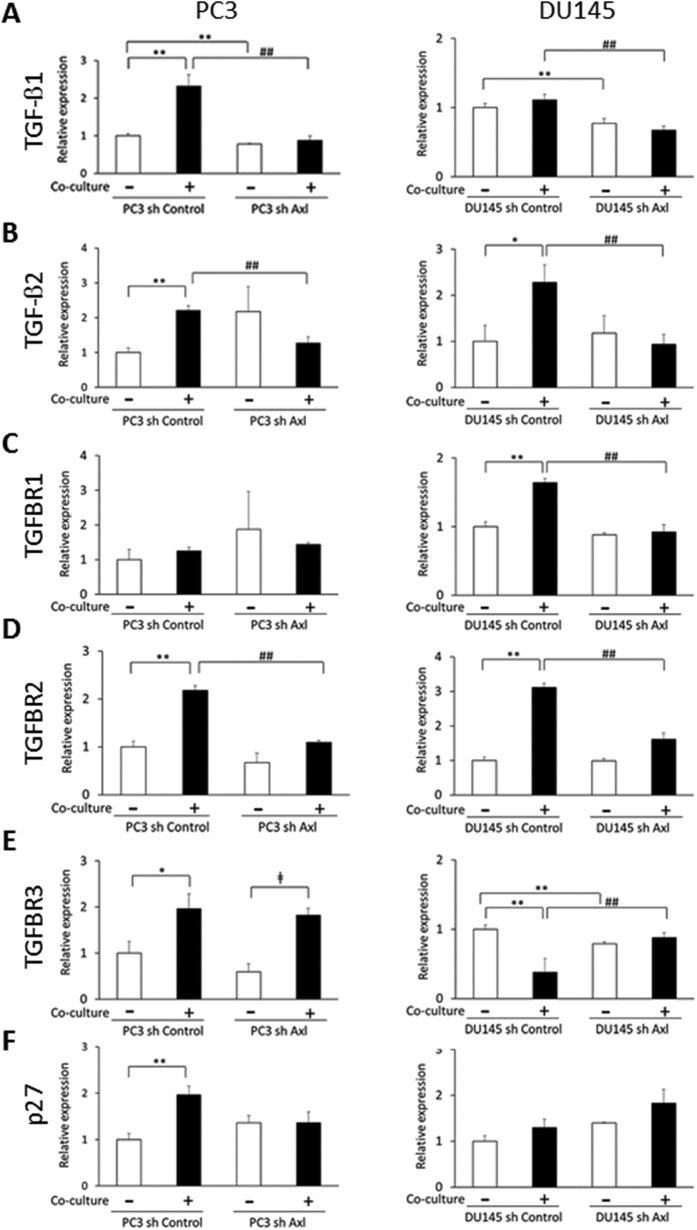
Relative expression of TGF-β ligands and receptors in PCa cells after 3 days of co-culture with MC3T3-E1 cells. (**A–F**) mRNA expression in PCa sh Control and PCa sh Axl cells measured by real time RT-PCR. *p < 0.05, **p < 0.01 compared to PCa sh Control cells cultured alone. ^##^p < 0.01 compared to PCa sh Control cells cultured with MC3T3. ^ǂ^p < 0.05 compared to PC3 sh Axl cells cultured alone.

To further explore the role that Axl plays in establishing cellular dormancy we examined the expression of TGF-β ligands and their receptors. TGF-β signaling has recently been shown to participate in dormancy of head and neck squamous cell carcinoma in the bone marrow^6^ and it is upregulated in DTCs from PCa patients with dormant disease for up to 18 years^6^. For these studies, mRNA expression was evaluated after 3 days of co-culture. We observed significant increases in TGF-β1 and TGF-β2 expression in PC3 cells (Fig. 4A,B). Similarly, the expression of the type II receptor (TGFBR2) and type III receptor (TGFBR3) were upregulated by the cancer cells in co-culture (Fig. 4D,E). Consistent with a dormancy signature of PCa cells after co-culture, p27, a potent cell cycle inhibitor and a downstream target of TGF-β signaling, was elevated in PC3 cells, suggesting that p27 may directly contribute to the induction of dormancy of PC3 cells (Fig. 4F). Surprisingly, both TGF-β1 and TGF-β2 were not increased in PC3 sh Axl cells after the co-culture (Fig. 4A,B). In addition, upregulation of TGFBR2 mRNA was significantly less in PC3 sh Axl cells compared to PC3 sh Control cells in the co-culture (Fig. 4D). Furthermore, the increase in p27 mRNA expression was completely abolished in PC3 sh Axl after the co-culture (Fig. 4F).

For DU145 cells, TGF-β2, TGFBR1 and TGFBR2 mRNA were dramatically upregulated after the co-culture (Fig. 4B–D). Axl knockdown inhibited the expression of TGF-β2 (Fig. 4B), and the enhanced expression of TGFBR1 and TGFBR2 mRNA were much less when compared to the sh Control cells in the co-culture (Fig. 4C,D). Unlike the PC3 cells, DU145 cells did not increase p27 mRNA in the co-culture after 3 days of the co-culture (Fig. 4F).

Cooperative cross-talk between tumor cells and members of the HSC niche are thought to play a significant role in regulating dormancy^15^,^23^. To explore this possibility, we examined the expression of TGF-β and Gas6 mRNA by MC3T3-E1 cells following the co-culture with PCa cells. Here it was observed that MC3T3-E1 cells recovered from the co-cultures with PC3 decreased their expression of TGF-β1, TGF-β2 and Gas6 (Fig. S4). As seen in the DU145 cells, MC3T3-E1 recovered from the co-cultures did not alter their expression of TGF-β1, TGF-β2 or Gas6 (Fig. S4). Knockdown of Axl in PCa cells did not influence TGF-β1, TGF-β2 or Gas6 expression in the co-cultured osteoblasts (Fig. S4).

### TGF-β is involved in the dormancy induction in PCa cells in the co-culture

**Figure 5:**
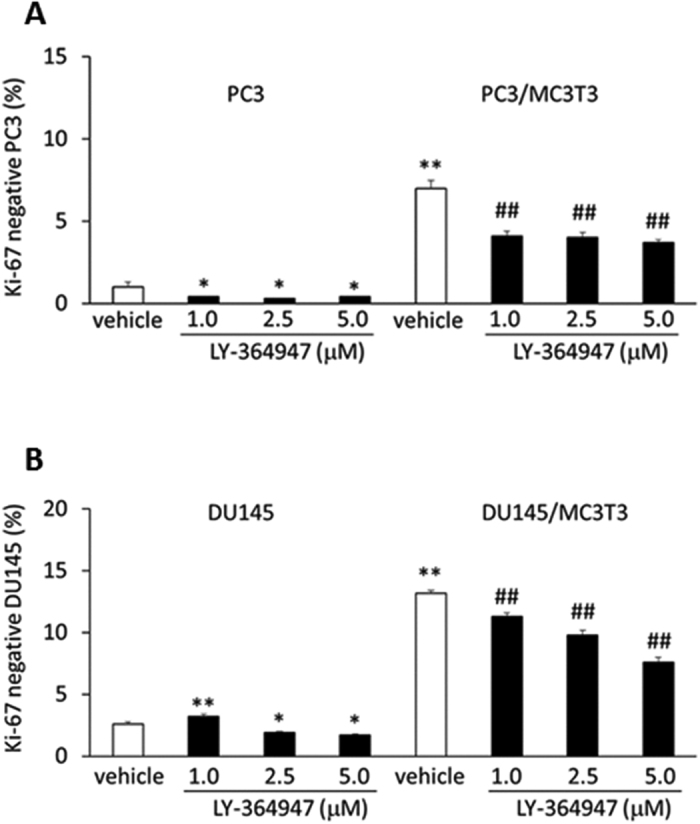
The effect of a TGFBR1 specific inhibitor (LY-364947) on Ki-67 expression by PCa after co-culture with MC3T3-E1 cells. (**A**) Ki-67 negative population in PC3 cells was evaluated after 4 days of co-culture with MC3T3-E1 cells. PC3 cells were treated with a TGFBR1 specific inhibitor, LY-364947 (Cat #: 2718, Tocris Bioscience, Minneapolis, MN) for 30 minutes at 37 °C, and then co-cultured with MC3T3-E1 cells. **p < 0.01 compared to PC3 cells cultured alone without the treatment of the inhibitor, ^##^p < 0.01 compared to PC3 cells co-cultured with MC3T3-E1 cells without the inhibitor. (**B**) Ki-67 negative populations in DU145 cells after 4 days of co-culture with MC3T3-E1 cells. *p < 0.05**, p < 0.01 compared to DU145 cells cultured alone in the absence of the inhibitor, ^##^p < 0.01 compared to DU145 cells co-cultured with MC3T3-E1 cells without the inhibitor.

We tested whether TGF-β is involved in the induction of dormancy of PCa cells by co-culture with MC3T3-E1 cells, using a TGFBR1 specific inhibitor, LY-364947. The inhibitor blocked the expansion of the Ki-67 negative populations in both PC3 and DU145 cells in the co-culture with MC3T3-E1 cells (Fig. 5A,B), suggesting that TGF-β signaling contributes to the induction of dormancy.

### Gas6 and Axl participate in the regulation of TGF-β family members and their receptors by PCa cells

**Figure 6:**
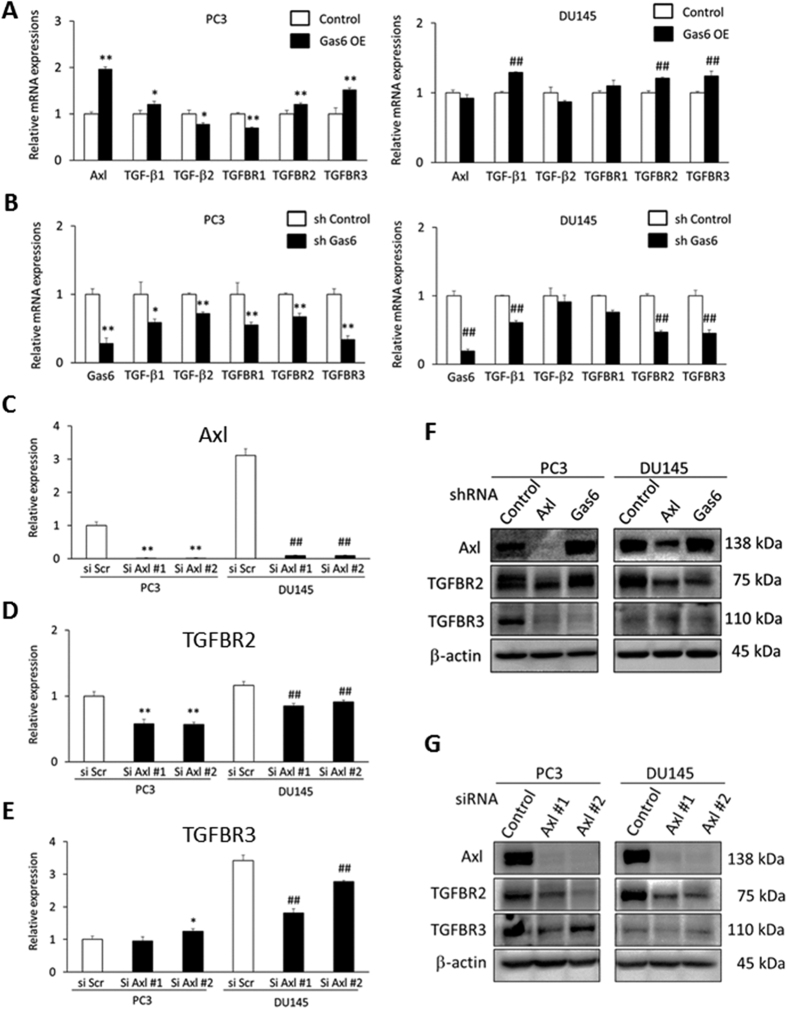
Gas6 and Axl expression are involved in the expression of TGF-β ligands and receptors in PCa cells. (**A**) mRNA expression in control and Gas6 overexpressed PCa cells. mRNA was extracted 2 days after serum starvation. *p < 0.05, **p < 0.01 compared to PC3 Control cells. ^##^p < 0.01 compared to DU145 Control cells. (**B**) mRNA expression in sh control and sh Gas6 knockdown PCa cells. mRNA was extracted after 6 days of culture in 0.5% FBS. Culture media was replaced at day3.*p < 0.05, **p < 0.01 compared to PC3 sh Control cells. ^##^p < 0.01 compared to DU145 sh Control cells. (**C–E**) mRNA expression in PCa cells 6 days after siRNA treatments targeting Axl. *p < 0.05, **p < 0.01 compared to si scramble control PC3cells, ^##^p < 0.01 compared to si scramble control DU145 cells. (**F,G**) Western blots showing protein expression levels of Axl, TGFBR2 and TGFBBR3 in PCa cells after the reduction of Axl or Gas6 by shRNA (**F**) or siRNA (**G**). Protein samples were loaded 6 days after siRNA treatments targeting Axl **(G)**.

Thus far our studies suggest that TGF-β signaling induces a dormant phenotype in PCa cells in co-culture with MC3T3-E1cells. While at the same time, reduction of Axl expression reduced the induction of PCa dormancy in the co-culture, which was associated with relatively lower expression of TGF-β ligands and TGFBR2 mRNA in PCa cells. Based on these results, we hypothesized that Gas6 may increase TGF-β ligand and TGFBR2 expression by signals generated through Axl. To address this, we examined the effect of Gas6 overexpression (Gas6 OE) or Gas6 knockdown (sh Gas6) on the mRNA expression of TGF-β ligands and their receptors in PCa cells (Fig. 6A,B). Gas6 protein expression levels in PCa Gas6 OE or PCa sh Gas6 were evaluated by ELISA (Fig. S5). Gas6 OE significantly increased the expression of TGF-β1, TGFBR2 and TGFBR3 both in PC3 and DU145 cells (Fig. 6A), while Gas6 knockdown significantly reduced their gene expression (Fig. 6B). We also stimulated PCa cells with recombinant Gas6. Recombinant Gas6 increased the expression of TGF-β1 and TGF-β2 in PC3 cells, and TGF-β2 in DU145 cells, however, their cognate receptor expression was not altered (Fig. S6). Next, we reduced Axl expression using two different siRNAs to confirm that Axl regulates TGFBR expression. Both Axl siRNAs (si Axl #1 and si Axl #2) successfully inhibited Axl mRNA expression in PC3 and DU145 cells (Fig. 6C). Here, TGFBR2 mRNA levels were reduced by siRNA targeting of Axl expression in both PC3 and DU145 cells (Fig. 6D).

TGF-β receptor expression was next examined at the protein level following Axl knockdown by sh RNA and siRNA (Fig. 6F,G). Western blots showed that stable knockdown of Axl by shRNA reduced TGFBR2 levels, and almost completely inhibited TGFBR3 expression in PC3 cells (Fig. 6F). In DU145 cells, sh Axl significantly reduced TGFBR2 levels while TGFBR3 was not detected (Fig. 6F). Similar findings were made when Axl expression was reduced by siRNA; TGFBR2 levels were dramatically reduced both in PC3 and DU145 cells (Fig. 6G), and TGFβR3 levels in PC3 were also reduced (Fig. 6G).

Next we examined the effect of reducing expression of the Axl ligand, Gas6 by shRNA. In PC3 cells, Gas6 reduction completely abolished the expression of TGFBR3 (Fig. 6F), whereas TGFBR2 levels were comparable with sh Control cells (Fig. 6F). In DU145 cells, Gas6 knockdown dramatically reduced TGFBR2 levels as did Axl knockdown (Fig. 6F).

Taken together, these results indicated that both Gas6 and Axl play a pivotal role in TGFBR expression. Remarkably, Axl knockdown inhibited TGFBR3 expression, whereas Mer or Tyro3 knockdown did not alter TGFBR3 expression at protein levels in PC3 cells (Fig. S7).

### Axl or Gas6 reduction diminishes the TGF-β2-mediated growth suppression

**Figure 7:**
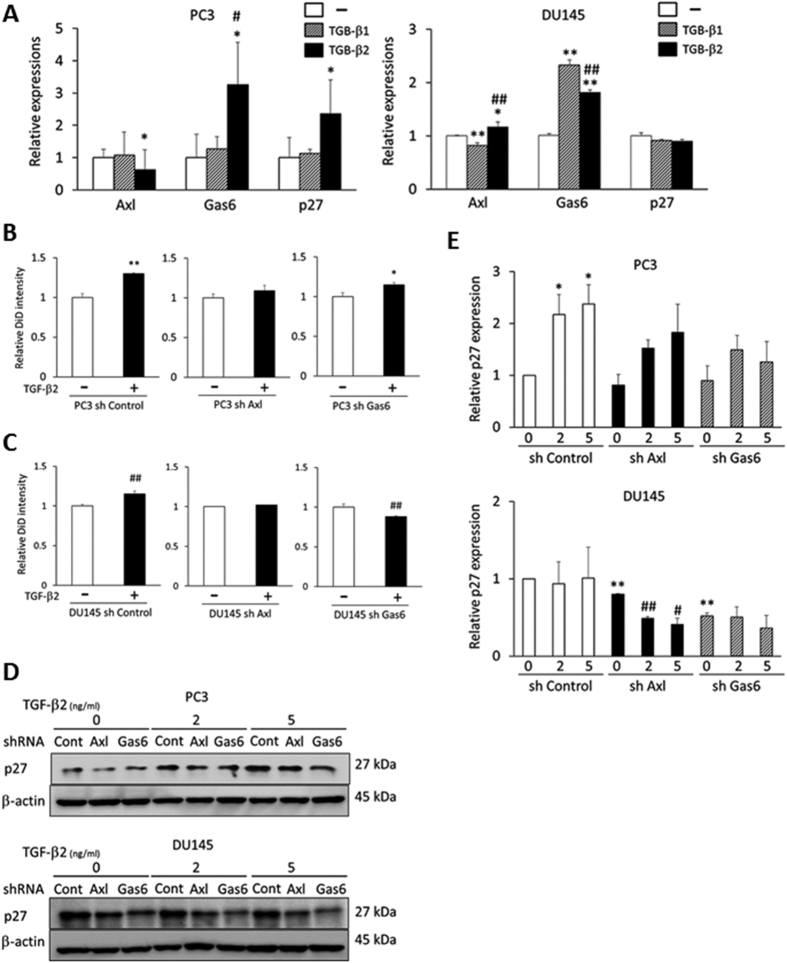
Axl or Gas6 is required for TGF-β2-induced growth suppression. (**A**) Relative mRNA levels in PCa cells were evaluated 24 hours after TGF-β1 (Cat #: 240-B/CF, R&D Systems, Minneapolis, MN) or TGF-β2 (Cat #: 302-B2/CF, R&D Systems, Minneapolis, MN) stimulation (both 2 ng/ml). *p < 0.05, **p < 0.01 compared to PCa cells without TGF-β stimulation, ^#^p < 0.05, ^##^p < 0.01 compared to PCa with TGF-β1 stimulation. (**B,C**) PCa cells were pre-labeled with DiD, and DiD retention was evaluated 6 days after TGF-β2 stimulation by flow cytometry. *p < 0.05, **p < 0.01 compared to PC3 cells without TGF-β2 treatments, ^##^p < 0.01 compared to DU145 cells without TGF-β2 treatments in each of sh Control, sh Axl or sh Gas6 cells. (**D**) Western blots showing p27 expression levels in PCa cells 3 days after TGF-β2 treatment. (**E**) Relative p27 expression levels evaluated by densitometry. P27 expression was normalized by β-actin expression. *p < 0.05, **p < 0.01 compared to PCa sh Control cells without TGF-β2 treatments. ^#^p < 0.05, ^##^p < 0.01 compared to DU145 sh Axl cells without TGF-β2 stimulation.

Our studies thus far have demonstrated that Gas6 and its receptor Axl play a significant role in the regulation of TGF-β ligands and their receptors. To determine whether TGF-β signaling might initiate a loop where the dormant phenotype is reinforced by autocrine signaling, we tested whether TGF-β2 vs TGF-β1 regulates the mRNA expression of Gas6, Axl and p27. TGF-β2, but not TGF-β1 significantly upregulated the expression of Gas6 and p27, while TGF-β2 downregulated Axl in PC3 (Fig. 7A). In DU145 cells, both TGF-β2 and TGF-β1 dramatically increased Gas6 expression, while TGF-β2, but not TGF-β1 significantly increased the expression of Axl (Fig. 7A). Unlike PC3 cells, expression of p27 was not altered by either of TGF-β1 or TGF-β2 in DU145 cells (Fig. 7A). These data suggest that TGF-β2 and Gas6 may induce a self-reinforcing loop to maintain a dormancy phenotype. Based on these results, we next hypothesized that Gas6-Axl axis may play a significant role on TGF-β-induced tumor cell growth suppression. To address this possibility, Axl or Gas6 knockdown in PCa cells were pre-labeled with DiD, and DiD retention was evaluated 6 days after TGF-β2 stimulation. TGF-β2 stimulation significantly increased the label retention in sh Control PCa cells (Fig. 7B,C), suggesting that PCa cell proliferation was inhibited by TGF-β2. The activity of TGF-β2 was nearly abolished by Axl or Gas6 knockdown, suggesting that both of Axl and Gas6 are required for the TGF-β2-mediated cell growth suppression in PCa cells (Fig. 7B,C).

To further explore the impact of Axl or Gas6 knockdown on the TGF-β-induced growth suppression, we examined p27 expression levels by Western blot after TGF-β2 treatment (Fig. 7D). In PC3 cells, p27 levels were significantly increased in sh Control cells 3 days after TGF-β2 stimulation, while p27 was not significantly enhanced by TGF-β2 stimulation both in sh Axl and sh Gas6 cells (Fig. 7D,E). In agreement with the RT-PCR results (Fig. 7A), p27 levels were not increased even after TGF-β2 treatment in DU145 cells (Fig. 7D,E), suggesting that another cell cycle inhibitor may work to inhibit proliferation after TGF-β2 stimulation. Interestingly, p27 levels were significantly lower both in sh Axl and sh Gas6 compared to sh Control DU145 cells (Fig. 7D,E). Furthermore, p27 was decreased by Axl reduction after TGF-β2 stimulation in DU145 cells (Fig. 7D,E), suggesting Gas6 and Axl is required to maintain p27 expression levels.

We also examined apoptosis of PCa cells after TGF-β2 treatment (Fig. S8). Interestingly, TGF-β2 significantly reduced apoptosis both in sh Control PC3 and DU145 cells, while Axl reduction almost completely impaired this activity of TGF-β2 (Fig. S8). Similarly, Gas6 knockdown diminished the effect of TGF-β2 on apoptosis in DU145 cells (Fig. S8). These results suggest that Gas6 and Axl are required for TGF-β2-induced signals which suppress apoptosis. These data also suggests that TGF-β2 suppresses PCa proliferation through the inhibition of cell cycle, rather than apoptosis induction.

### Axl reduction suppresses PCa cellular dormancy in bone marrow

**Figure 8:**
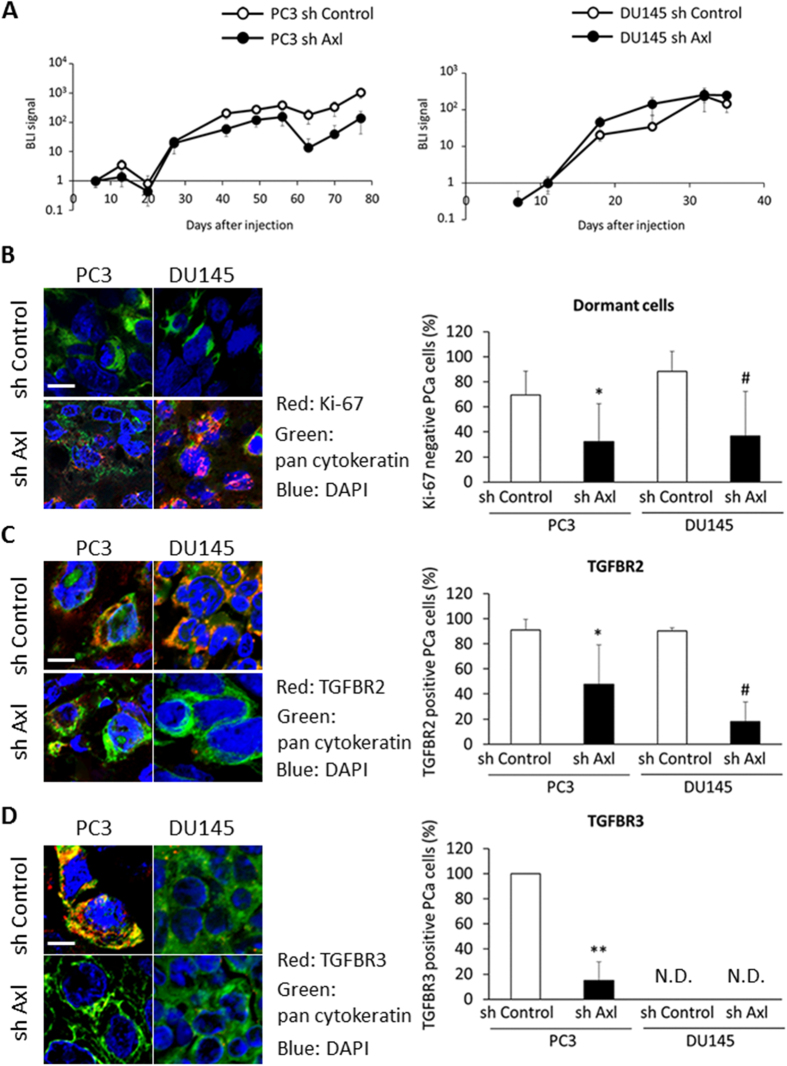
Axl reduction in PCa cells suppresses the cellular dormancy of PCa cells in bone marrow. (**A**) Tumor growth monitored by bioluminescence imaging (BLI) in marrow. PCa sh Control and sh Axl cells labeled with luciferase were injected into tibiae of SCID mice (n = 5). (**B**) Immunohistochemistry showing Ki-67 expression in PCa sh Control and PCa sh Axl cells in BM. PCa cells were recognized by pan cytokeratin with green color, and Ki-67 was shown with red color. Scale bar, 20 μm. Ki-67 negative and positive PCa cells were counted and the percentage of Ki-67 negative PCa cells was shown. *p < 0.05 compared to PC3 sh Control cells. ^#^p < 0.05 compared to DU145 sh Control cells. (**C**) Immunohistochemistry showing TGFGBR2 expression in PCa sh Control and PCa sh Axl cells in BM. PCa cells were recognized by pan cytokeratin with green color, and TGFBR2 was shown with red color. Scale bar, 10 μm. TGFBR2 positive and negative PCa cells were counted and the percentage of TGFBR2 positive PCa cells was shown. *p < 0.05 compared to PC3 sh Control cells. ^#^p < 0.05 compared to DU145 sh Control cells. (**D**) Immunohistochemistry showing TGFGBR3 expression in PCa sh Control and PCa sh Axl cells in BM. PCa cells were recognized by pan cytokeratin with green color, and TGFBR3 was shown with red color. Scale bar, 10 μm. TGFBR3 positive and negative PCa cells were counted and the percentage of TGFBR3 positive PCa cells was shown. **p < 0.01 compared to PC3 sh Control cells. N.D. means not detected.

To determine whether Axl expression is involved in PCa cellular dormancy or survival *in vivo*, we injected PCa sh Axl cells labeled with luciferase into the tibiae of SCID mice. Tumor growth was monitored by bioluminescence images (BLI) (Fig. 8A), and bone sections were examined after tumor lesions were confirmed. Fewer Ki-67 negative PCa cells (dormant) were present in the marrow of animals injected with sh Axl cells compared to sh Control cells (Fig. 8B). Likewise, TGFBR2 expression was suppressed in PCa sh Axl cells (Fig. 8C), and TGFBR3 expression was reduced by Axl reduction in PC3, although TGFBR3 was not detected in DU145 cells (Fig. 8D). Furthermore, terminal deoxynucleotidyl transferase dUTP Nick-End Labeling (TUNEL) assay showed that apoptotic cells appear to be augmented in PCa sh Axl cells in BM (Fig. S9). These results suggest that Axl plays a significant role in the regulation of TGFBR expression, tumor cell growth and survival in DTCs found in the marrow.

## Discussion

Our previous studies suggest that osteoblasts play a key role in inducing and/or maintaining PCa dormancy in the marrow^15^. We assumed that part of the mechanism used by osteoblasts to regulate dormancy is through the secretion of Gas6 and activation of its receptor, Axl^17^. However, we did not have evidence which directly links Axl to dormancy induction, although we observed Axl expression is associated with PCa cell dormancy status in the bone marrow^20^.

In the present study, we provide evidence that Axl expression regulates cellular dormancy of PCa cells stimulated by osteoblasts (Fig. 3A–C). Interestingly, the co-culture with osteoblasts elevates the expression of both TGF-β2 and TGFBR2 in PCa cells (Fig. 4B,D), which contributes to dormancy induction of PCa cells. In fact, diminished Axl expression impairs TGF-β2 and TGFBR2 expression (Fig. 4B,D). Furthermore, loss of Axl expression limited the impact of TGF-β2 on inhibiting PCa cell growth (Fig. 7B,C). Together these findings suggest that Axl is a crucial regulator of dormancy induced by TGF-β signaling in PCa cells. Therefore this is the first report to our knowledge to demonstrate that Gas6/Axl signals regulates TGFBR expression, and also the Gas6/Axl axis is required for TGF-β2-mediated growth suppression of PCa cells.

**Figure 9:**
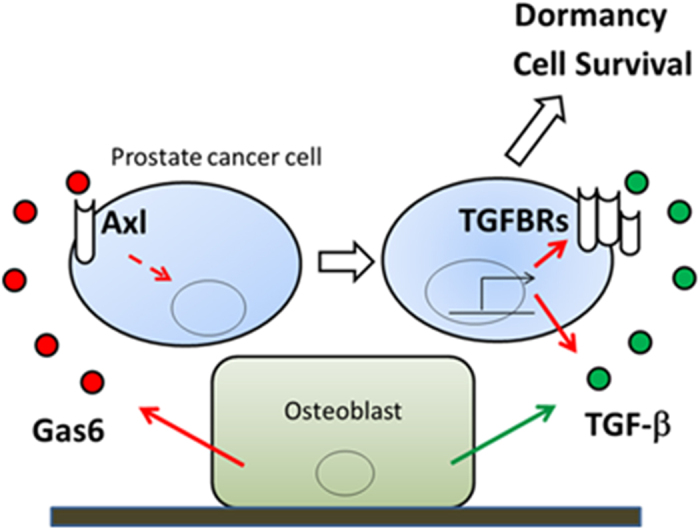
A model of osteoblast-mediated PCa cellular dormancy through Gas6 and TGF-β signaling in marrow. Gas6 produced by osteoblasts binds to Axl expressed by disseminated PCa cells, and its signaling induces expression of both TGF-β ligands (TGF-β1 and TGF-β2) and their receptors (TGFBR2 and TGFBR3). Subsequently, autocrine and paracrine TGF-β signaling induces PCa dormancy.

Axl has complex and diverse roles in cell biology. Axl regulates cell proliferation, survival, epithelial and mesenchymal transition (EMT), migration, and innate immunity^19^,^24^. Gas6, the primary ligand of Axl, induces the phosphorylation of Axl, which causes the activation of cell signal transducers including MAPK, PI3 kinase, AKT, and mTOR, and induces or inhibits downstream target gene expression. Recently, it was reported that Gas6-Axl signaling increases TGF-β1 expression in mesenchymal hepatocellular carcinoma (HCC) cells via the activation of c-Jun N-terminal kinase (JNK), which promotes Smad3 function^25^. Our results that Gas6 increases TGF-β1 expression in PCa cells are consistent with that observation (Figs 6A and S6). Our results also show that Gas6 signaling increases TGF-β2 expression (Fig. S6), which induces dormancy in head and neck squamous cell carcinoma (HNSCC) and breast cancer cells^6^. TGF-β2 was also recently found upregulated in dormant DTCs from PCa patients free of disease for up to 18 years^14^,^26^. Further, PCa cells isolated from prostate cancer patient derived xenografts must downregulate TGF-β2 to exit dormancy when grown with BM stromal feeder layers^27^. TGF-β2 and TGFBR3 were found upregulated in dormant basal type DTCs from breast cancer patient-derived xenograft (PDX) studies^28^, arguing that this pathway might regulate dormancy in multiple tissues and epithelial cancers. Importantly, Axl maintained basal levels of both TGFBR2 and TGFBR3 expression (Fig. 6F,G). TGFBR3 was found to be essential for TGF-β2-induced dormancy and loss of this receptor is commonly associated with bone metastasis^6^,^29^,^30^. Taken together, these results support a model of osteoblast-mediated PCa cellular dormancy through Gas6 and TGF-β signaling in marrow (Fig. 9); Gas6 produced by osteoblasts binds to Axl expressed by disseminated PCa cells, and its signaling induces expression of both TGF-β ligands (TGF-β1and TGF-β2) and their receptors (TGFBR2 and TGFBR3). Subsequently, autocrine and paracrine TGF-β signaling induces PCa dormancy.

TGF-β is involved in many steps of tumor metastases. At primary sites, some tumor cells acquire invasive properties to penetrate the walls of lymphatic or blood vessels, and circulate through the bloodstream to local tissues including bone. It is well established that TGF-β facilitates the tumor cell invasion through the induction of EMT^13^. After tumor cells reach the bone marrow, TGF-β induces the dormancy and supports the survival of tumor cells even in the presence of current chemotherapy drugs^31^,^32^,^33^. Later, TGF-β released from the bone matrix through osteoclastic activity has been shown to create a ‘vicious cycle’ by stimulating tumor growth and activating further osteolysis^34^. TGF-β ligands bind their type II receptors (TGFBR2), which recruits type I receptors (TGFBR1), and the activated TGFBR1 phosphorylates its intracellular substrates, Smad2 and Smad3, on their C-terminal SSXS motif 35. The phosphorylated Smads form complexes with Smad4, and these complexes accumulate in the nucleus and regulate gene transcription, including p27, which is linked to dormancy induction in many tumor cells. TGFBR2 has been shown to act as a tumor-suppressor gene^36^,^37^,^38^. TGFBR2 is involved in the regulation of tumor cell proliferation, and human prostate tumors often escape from TGF-β-mediated growth inhibition via downregulation of TGFBR2^39^,^40^,^41^,^42^. Type III receptors (TGFBR3), which belongs to co-receptors of the TGF-β pathway, preferentially binds TGF-β2, and can enhance the inhibitory effects of TGF-β2 on cell proliferation^6^.

Interestingly, the co-culture conditions dramatically increased TGFBR2 expression in both PC3 and DU145 cells. Inhibition of TGF-β signaling by the specific TGFBR inhibitor significantly suppressed PCa cell dormancy induction in our co-culture conditions (Fig. 5A, B), indicating that TGF-β is one of the key regulators of dormancy in this condition, and suggesting TGFBR2 expression promotes TGF-β-mediated tumor cell growth inhibition. Curiously, Axl knockdown dramatically reduces TGFBR2 expression (Fig. 6D,F,G). In addition, stable knockdown of Axl also downregulates TGFBR3 in PC3 cells, which is required for induction of tumor cell dormancy by TGF-β2 and also essential for TGF-β2 presentation to the Type I and Type II receptpors^6^, whereas Mer or Tyro3 knockdown does not alter TGFBR3 expression (Fig. S7).

To address what induces TGFBR expression, we stimulated PCa cells with recombinant human Gas6. Although recombinant Gas6 increased TGF-β1 or TGF-β2 expression, TGFBR2 or TGFBR3 expression levels were not altered after the stimulation (Fig. S6). However, Gas6 overexpression increases both TGFBR2 and TGFBR3 expression (Fig. 6A). These observations imply that recombinant Gas6 which we used was not able to induce TGFBRs as efficiently as perhaps endogenously expressed Gas6. It is well appreciated that the activity of Gas6 will vary depending on post-translational processing including gamma-carboxylation, therefore, conditions in which Gas6 is expressed and purified are critical^43^. Although further studies are required to identify the precise ligands which bind Axl and regulate TGFBR expression, our results strongly suggest that Axl regulates TGFBR2 or TGFBR3 expression levels which are required for TGF-β-mediated dormancy induction in PCa cells.

Once tumors arrive in the marrow, a potentially harsh environment for epithelial cells, the activation of critical survival strategies are likely to be required for survival. One of the most important aspects of Axl function is that Axl supports survival of tumor cells through the activation of PI3K-Akt signaling^44^,^45^. Previously, it was shown that Axl prevented apoptosis after TGF-β1stimulation in HCC cells^25^. We asked if the reduction of Axl alters the apoptosis in PCa cells after TGF-β2 stimulation. TGF-β2 significantly reduced apoptosis in PCa cells, while Axl reduction impaired the anti-apoptotic activity of TGF-β2 (Fig. S8). TGF-β signaling supports the survival of tumor cells^11^,^31^,^32^,^33^, and our results suggest that Axl is required for TGF-β-mediated survival signals. Furthermore, in our animal models, Axl reduction increased apoptotic PCa cells in the bone marrow (Fig. S9), while Axl knockdown PCa cells showed relatively higher expression of proliferation marker, Ki-67 (Fig. 8B). Taken together, our results suggest that Axl is a crucial regulator for not only proliferation, but also tumor survival in bone marrow.

Bone metastases are derived from DTCs that enter into cellular dormancy in bone marrow. Dormant tumor cells are resistance to apoptotic signals even in the presence of conventional chemotherapeutic drugs, which prevents elimination of DTCs by chemotherapy. Importantly, TGF-β induces dormancy and supports the survival of DTCs, which is enhanced by the induction of TGF-β and TGFBR expression by Gas6/Axl signaling in DTCs. Therefore, blocking Axl functions may potentially lead to a suppression of TGF-β-mediated dormancy. Thus, Axl signaling should be considered as a potential therapeutic focus to target DTCs in bone to prevent bone metastases.

## Methods

### Cell culture

The human prostate cancer cell lines, PC3 (Cat #: CRL-1435) and DU145 (Cat #: HTB-81), and murine pre-osteoblastic cell line, MC3T3-E1 (Cat #: CRL-2593) were obtained from the American Type Culture Collection. All prostate cancer cell lines were routinely grown in RPMI 1640 (Life Technologies, Carlsbad, CA, USA) supplemented with 10% (v/v) fetal bovine serum (Invitrogen), 1% (v/v) penicillin-streptomycin (Invitrogen) and maintained at 37 °C, 5% CO2, and 100% humidity. MC3T3-E1 cells were grown in minimal essential medium (MEM) alpha (Life Technologies, Carlsbad, CA, USA) containing 10% (v/v) fetal bovine serum (Invitrogen), 1% (v/v) penicillin-streptomycin (Invitrogen) and maintained at 37 °C, 5% CO2, and 100% humidity.

### Co-culture *in vitro*

MC3T3-E1 cells (2 × 10^5^ cells) were seeded in 12-well plates in MEM-alpha containing 10% (v/v) fetal bovine serum, and 24 hours later, PCa cell lines (2 × 10^4^ cells) were plated on the subconfluent MC3T3-E1 cells. When using 100 mm dishes, PCa cell lines (2 × 10^5^ cells) were plated on the 80% confluent MC3T3-E1 cells.

### Luciferase based proliferation assay

Luciferase activity was measured by a Luciferase assay system (Cat #: E1910, Promega, Madison, WI). Cells were rinsed with phosphate-buffered saline (PBS) once, and treated with the lysis buffer provided by the kit. Supernatants were collected by centrifugation, mixed with Luciferase assay substrates, and relative light units (RLU) was measured by Monolight 2010 luminometer (Analytic Luminescent Laboratories, San Diego, CA).

### Flow cytometry

The flow cytometric analyses and fluorescence-activated cell sorting (FACS) were performed on a FACS Aria dual-laser flow cytometer (Becton Dickinson, Franklin Lakes, NJ) and data were analyzed with DIVA software (Becton Dickinson). BD cytometer setup & tracking beads (BD Biosciences, Cat #: 642412) were used for the daily instrument standardization and validation. Sorting calibration was performed before each sort by drop-delay using Accudrop beads (BD Biosciences, Cat #: 345249). Sorting of cells was performed using a 70 μm nozzle at 70 psi in purity mode.

### BrdU incorporation

Cells were cultured in the presence of 10 μM BrdU for 30 minutes, and then BrdU was detected following the manufacturer’s protocol (BrdU Labeling kit, Cat #: 552598, BD Biosciences, San Jose, CA). Cells were also stained with a FITC-anti-HLA-ABC antibody (clone W6/32: Cat #: 311404, BioLegend, San Diego, CA). PCa cells were gated as HLA-positive populations and BrdU-positive cells were detected by a FACS Aria dual-laser flow cytometer.

### Cell labeling with DiD dye

PCa cell lines were stained with DiD dye (Cat #: v22887, Molecular Probes, Eugene, OR). Cells (1 × 10^6^ cells/ml) were incubated with DiD dye (0.5 μM) in serum-free conditions at 37 °C for 20 min, and then were washed three times with serum-free medium. The intensity of DiD was analyzed on a FACS Aria dual-laser flow cytometer and data were analyzed with DIVA software.

### Ki-67 staining

Cells were fixed with cold 70% ethanol, and stained with an APC conjugated anti-human Ki-67 antibody (Cat #: 350513, Biolegend) in PBS containing 2% FBS for 30 minutes at room temperature. When PCa cells were co-cultured with murine MC3T3-E1cells, cells were stained with a FITC-anti-HLA-ABC antibody (clone W6/32: Cat #: 311404, BioLegend) to identify human PCa cells before fixation. Ki-67 negative PCa cells were examined using a FACS Aria dual-laser flow cytometer.

### TAM receptors expression examined by flow cytometry

Cells were dissociated by the treatment with 10 mM EDTA, and resuspended in PBS containing 2% FBS. Cells were stained with anti-human Axl antibody (Cat #: FAB154P, R&D), anti-human Mer antibody (Cat #: FAB8912P, R&D), and anti-human Tyro3 antibody (Cat #: FAB859P, R&D) for 30 minutes at 4 °C. TAM receptors expression was examined using a FACS Aria dual-laser flow cytometer.

### Stable knockdown of TAM receptors and Gas6

Stable knock down of the TAM receptors (Tyro3, Axl, Mer) or Gas6 in PCa cell lines were established by lentiviral transduction^46^. Lentiviruses were constructed by the University of Michigan Vector Core using pGIPZ lentiviral vectors containing a shRNA targeting each of TAM receptors individually. The lentivirus infections were performed in the presence of 5 μg/mL polybrene for 48 hours, and then the cells were cultured in medium containing 10 μg/mL puromycin for 2 weeks to generate stable cell lines. The knockdown of each TAM receptors was verified by Western blot (Fig. S3). The knockdown of Gas6 was confirmed by ELISA (Fig. S5).

### Axl transient siRNA knockdowns

Two siRNAs targeting Axl (Cat #: 4390824, S1846 and S1847) and control siRNA (si Control) (Cat #: 4390843) were purchased from Thermo-Fisher Scientific. Transient transfection in PC3 and DU145 cells was performed using 200 nM of each siRNA with Lipofectamine RNAiMAX reagent (Cat #: 13778-150, Invitrogen).

### Gas6 overexpression in PCa cells

Human GAS6 overexpression plasmid vector (Ex-Z6826-Lv105) and control vector, (Ex-NEG-Lv105) (GeneCopia, Rockville, MD) were packaged with lentivirus at the University of Michigan Vector Core Facility. Lentiviral human GAS6 vector or control vector were infected into PCa cells (PC3 and DU145). Infected cells were selected for 7 days in media containing 1 μg/ml puromycin. Human GAS6 overexpression was verified by ELISA (Fig. S5).

### Immunostaining

Cells were fixed and permeabilized with Perm/Wash Buffer (Cat. # 554723, BD Biosciences). Samples were blocked with Image-iT FX signal enhancer for 30 min and incubated for 2 hours at room temperature with primary antibodies combined with reagents of Zenon Alexa Fluor 488 (green) or 555 (red) labeling kit (Invitrogen). To detect Ki-67 in PCa cells co-cultured with MC3T3-E1 cells *in vitro*, human Ki-67 (cat. # ab15580, Abcam) and HLA-ABC (Cat. # 311402, BioLegend) antibodies were used as primary antibodies. To detect Ki-67 in PCa cells in bone marrow sections, human Ki-67 (cat. # ab15580, Abcam) and pan cytokeratin (cat. # ab867364, Abcam) antibodies were used as the primary antibody. To detect human TGFBR2 and TGFBR3, human TGFBR2 (cat. # ab61213, Abcam) and human TGFBR3 (cat. # ab78421, Abcam) antibodies were used as the primary antibody. After washing with PBS, the slides were mounted with ProLong Gold antifade reagent with DAPI (Invitrogen). Images were taken with Nikon A-1-B confocal microscope.

### Detection of apoptotic PCa cells by terminal deoxynucleotidyl transferase (TdT)-mediated dUTP nick end labeling (TUNEL) assay

Cell Meter TUNEL Apoptosis Assay Kit (Cat #: 22844, AAT Bioquest, Sunnyvale, CA) was used to detect apoptotic PCa cells in mice bone marrow sections following manufacturer directions. Briefly, sections were fixed with 4% formaldehyde, and rinsed with PBS. The sections were incubated with the kit reaction mixture for 1 hour at 37 °C. After washing with PBS, the samples were treated with pan cytokeratin (cat. # ab867364, Abcam) antibodies combined with reagents of Zenon Alexa Fluor 488 (green) labeling kit (Invitrogen) to detect PCa cells. The slides were mounted with ProLong Gold antifade reagent with DAPI (Invitrogen). Images were taken with Nikon A-1-B confocal microscope.

### Apoptosis measured by Annexin V binding assay

Apoptotic cells were detected by flow cytometry (FACS Aria dual laser flow-cytometer, Becton Dickinson, Mountainview, CA) using PE Annexin V Apoptosis Detection Kit I (cat. 559763, BD Biosciences, San Jose, CA).

### RNA Extraction and Real-Time RT-PCR

Total RNA was isolated using RNeasy Mini Kit (QIAGEN, Valencia, CA). First-strand cDNA was synthesized in a 20 μL reaction volume using 1 μg of total RNA. RT products were analyzed by real-time RT-PCR in TaqMan^®^ Gene Expression Assays (Applied Biosystems, Foster City, CA), using human-specific or mouse-specific TaqMan MGB probes (Applied Biosystems). PCR products were detected as an increase in fluorescence using an ABI PRISM 7700 instrument (Applied Biosystems). TaqMan MGB probes (Applied Biosystems) were as follows: human-specific probes; Gas6 (Hs01090305_m1), Tgfb1(Hs00998133_m1), Tgfb2(Hs00234244_m1), TGFBR1(Hs00610318_m1), TGFBR2 (Hs00559660_m1), TGFBR3 (Hs00234257_m1), p27 (CDKN1B)(Hs00153277_m1), Axl (Hs01064445_m1), Tyro3(Hs03986773_m1), MerTK(Hs00179024_m1).

Mouse-specific probes; Gapdh (Mm99999915_g1), Gas6 (Mm00490378_m1), Tgfb1(Mm01178819_m1), Tgfb2 (Mm03024009_m1).

β-actin (Hs01060665_g1), Gapdh (Hs99999905_m1) or Gapdh (Mm99999915_g1) were used as internal controls for the normalization of target gene expression in human PCa cells or murine MC3T3-E1 cells, respectively.

### Western blots

PCa cells were cultured in RPMI 1640 with 10% FBS and 1% P/S. Whole cell lysates were separated on 4–20% Tris-Glycine gel and transferred to PVDF membranes. The membranes were incubated with 5% bovine serum albumin (Cat. # A2153, Sigma) for 1 hour and incubated with primary antibodies overnight at 4 °C. Primary antibodies used were as follows: human Axl (1:1,000 dilution, Cat. #4939, Cell Signaling), human p27 (1:1,000 dilution, Cat. #3686, Cell Signaling), human TGFBR2 (1:1,000 dilution, Cat. # ab184948, Abcam), human TGFBR3 (1:1,000 dilution, Cat. # 2519, Cell Signaling). Blots were incubated with peroxidasecoupled anti-rabbit IgG secondary antibody (Cat. # 7074, 1:2,000 dilution, Cell Signaling) for 1 hour, and protein expression was detected with SuperSignal West Dura Chemiluminescent Substrate (Cat. #34075, Thermo Scientific, Rockford, IL). Membranes were reprobed with monoclonal anti-β-actin antibody (1:2,000 dilution; Cat. # 4970, Cell Signaling) to control for equal loading.

### ELISA

An antibody sandwich ELISA was used to evaluate Gas6 expression in the conditioned media and cell lysates by the following the directions of manufacturer (Cat. #DY986 and DY885, R&D Systems, Minneapolis, MN). Gas6 levels were normalized to total cell numbers.

### Animal experiments

All experimental animal procedures were performed in compliance with the institutional ethical requirements and approved by the University of Michigan Institutional Animal Care and Use Committee (IACUC). GFP/luciferase-expressing PCa sh Control or PCa sh Axl cells (2 × 10^5^ cells) were suspended in 100 μl of PBS and injected into male CB.17. SCID mice (6–8 weeks of age, Charles River, Wilmington, MA) by intratibial (i.t.) injection. Tumor growth was monitored by bioluminescence images (BLI). At the experimental endpoint, mice were sacrificed, and the tibiae which PCa cells were injected were collected for further histological analysis.

### Statistical Methods

All numerical data are expressed as mean ± standard deviation unless specified otherwise. Two-tailed, unpaired Student’s *t*-test was used for data analysis, with p < 0.05 considered to be statistically significant.

## Additional Information

**How to cite this article**: Yumoto, K. *et al*. Axl is required for TGF-β2-induced dormancy of prostate cancer cells in the bone marrow. *Sci. Rep.* **6**, 36520; doi: 10.1038/srep36520 (2016).

**Publisher’s note:** Springer Nature remains neutral with regard to jurisdictional claims in published maps and institutional affiliations.

## Supplementary Material

Supplementary Information
