# Supplementary material for: Axl is required for TGF-β2-induced dormancy of prostate cancer cells in the bone marrow
Source: Sci Rep. 2016 Nov 7;6:36520. doi: 10.1038/srep36520 (PMC5098246; doi:10.1038/srep36520)
Supplement: Supplementary Information [file srep36520-s1.pdf]

Supplementary Information to

"Axl is required for TGF-β2-induced dormancy of prostate cancer cells in the bone marrow"

Kenji Yumoto, Matthew R. Eber, Jingcheng Wang, Frank C. Cackowski, Ann M. Decker, Eunsohl Lee, Ana Rita Nobre, Julio A. Aguirre-Ghiso, Younghun Jung and Russell S. Taichman

A

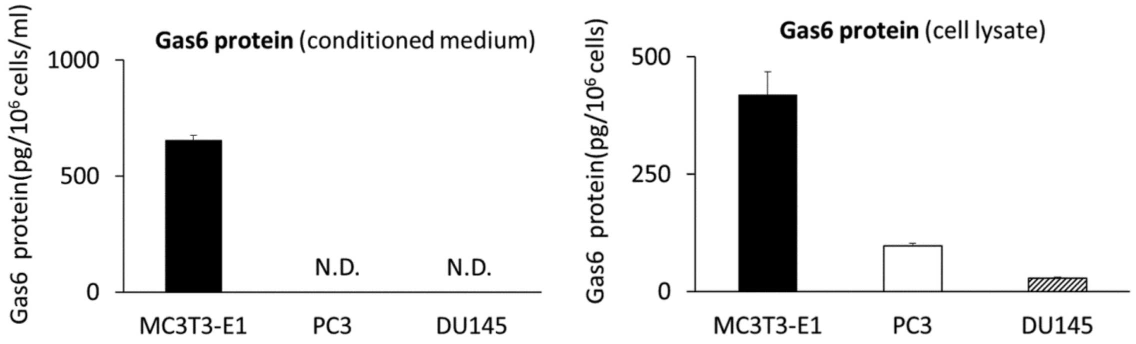

B

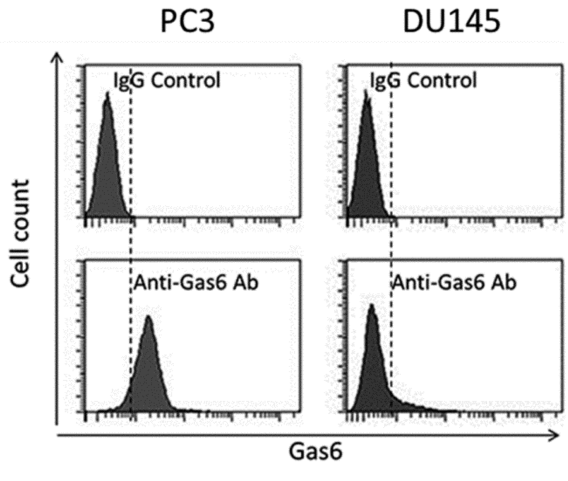

Figure S1.Gas6 protein expression. (A) Gas6 protein levels in the conditioned medium and cell lysates from MC3T3-E1, PC3 and DU145 cells were evaluated by ELISA. (B) Detection of cell surface expression of Gas6 by PCa cells.

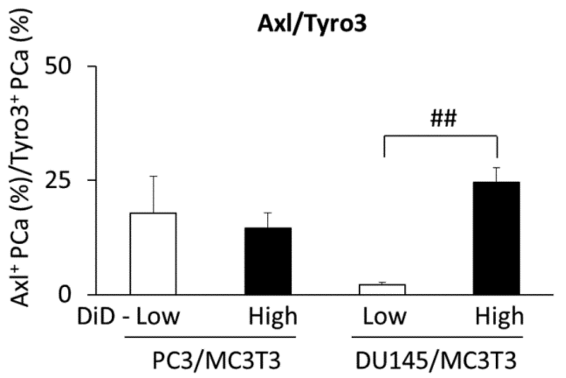

Figure S2: Cell surface expression levels of Axl and Tyro3 ratio (Axl/Tyro3). Cell surface expression of Axl and Tyro3 was examined between DiD-low and DiD-high PCa cells cultured in the presence of MC3T3-E1 cells by flow cytometry. ##p<0.01 compared to DiD-low DU145 cells cultured in the presence of MC3T3-E1 cells.

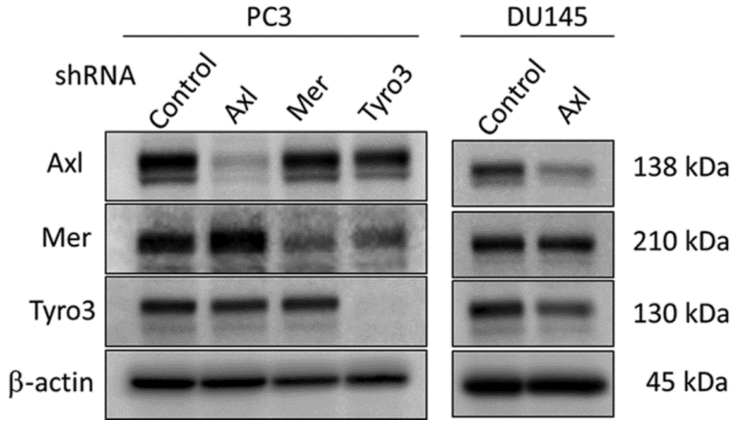

Figure S3: The efficiency of TAM receptors knockdown by shRNA evaluated by western blot.

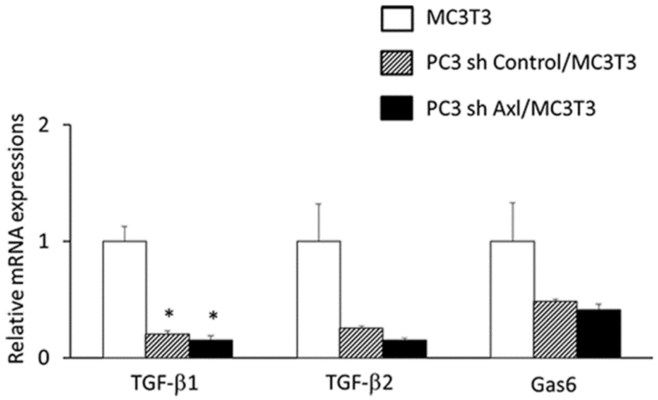

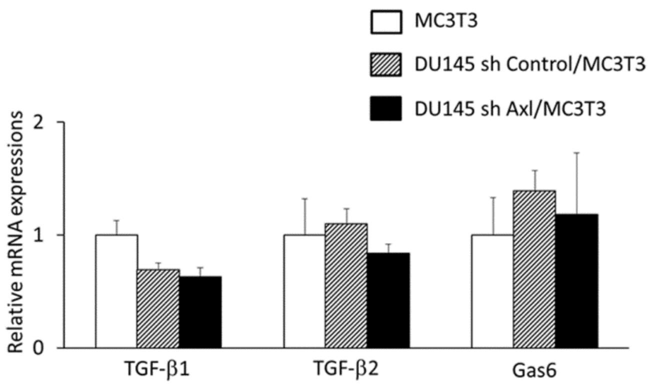

Figure S4: mRNA expression in MC3T3-E1 cells with or without PCa sh Control or PCa sh Axl cells. *$p<0.05$ compared to MC3T3-E1 cells cultured alone.

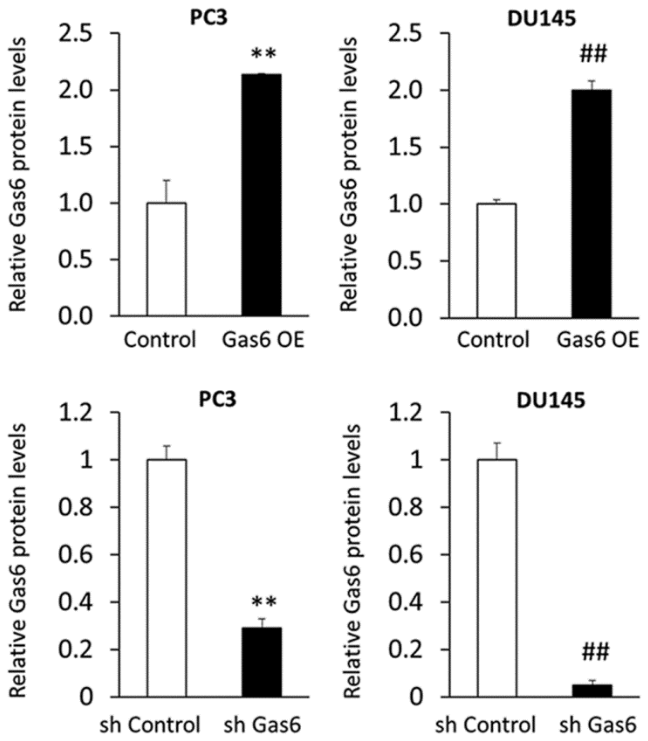

Figure S5: Gas6 protein expression levels in Gas6 overexpression PCa cells (PCa Gas6 OE) and Gas6 sh knockdown PCa cells (PCa sh Gas6). **$p<0.01$ compared to Control PC3 cells. ##$p<0.01$ compared to Control DU145 cells.

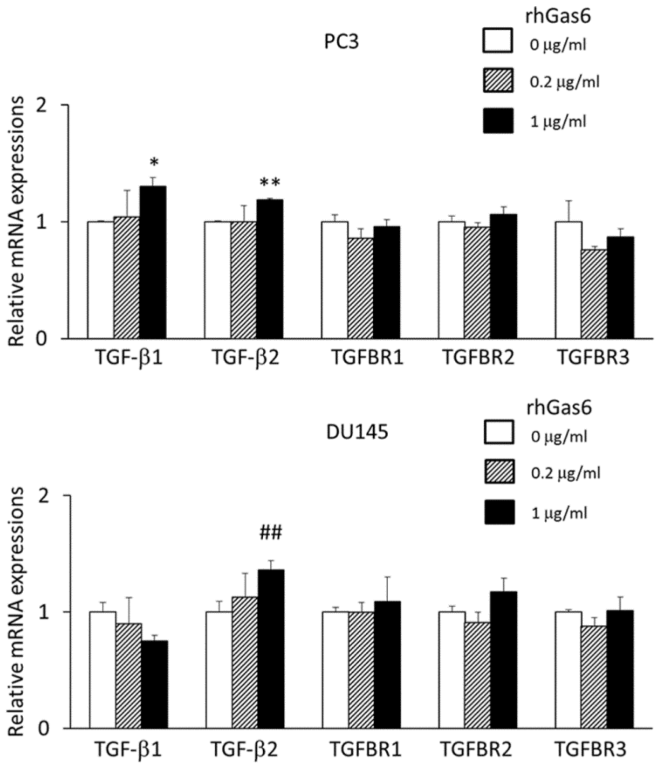

Figure S6: mRNA expressions in PCa cells measured by RT-PCR 2 days after recombinant human Gas6 (Cat #: AF885, R&D Systems, Minneapolis, MN) stimulation. *$p<0.05$, **$p<0.01$ compared to PC3 cells without the stimulation. ##$p<0.01$ compared to DU145 cells without the stimulation.

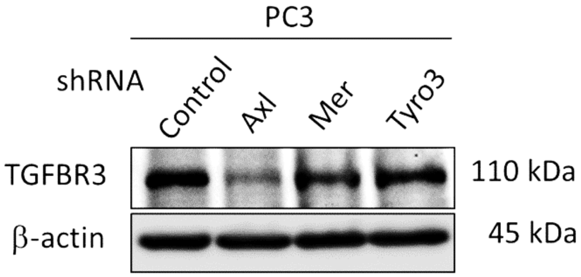

Figure S7: A western blot showing TGFBR3 protein expression in each of PC3 sh Control, sh Axl, sh Mer or sh Tyro3 cells.

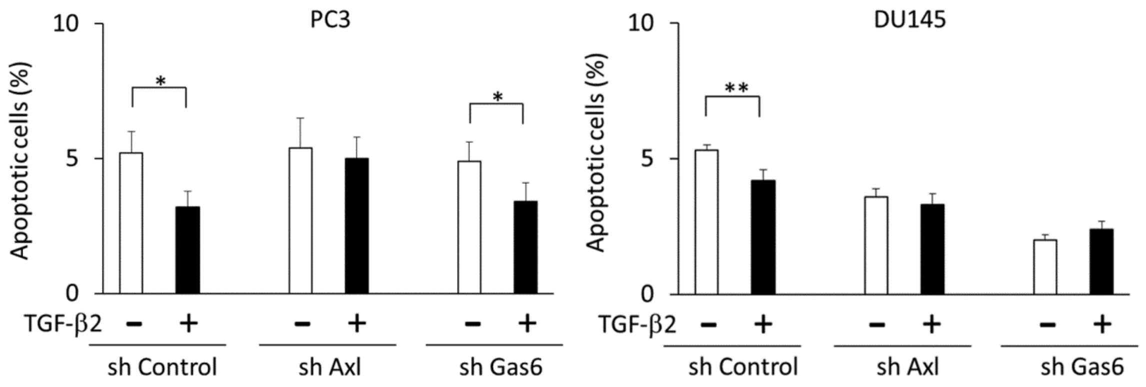

Figure S8: Apoptosis evaluated by annexin V binding to PCa cells 3 days after TGF-β2 (5ng/ml) treatment. *p<0.05, **p<0.01 compared to PCa cells without TGF-β2 treatments in each of sh Control, sh Axl or sh Gas6 cells.

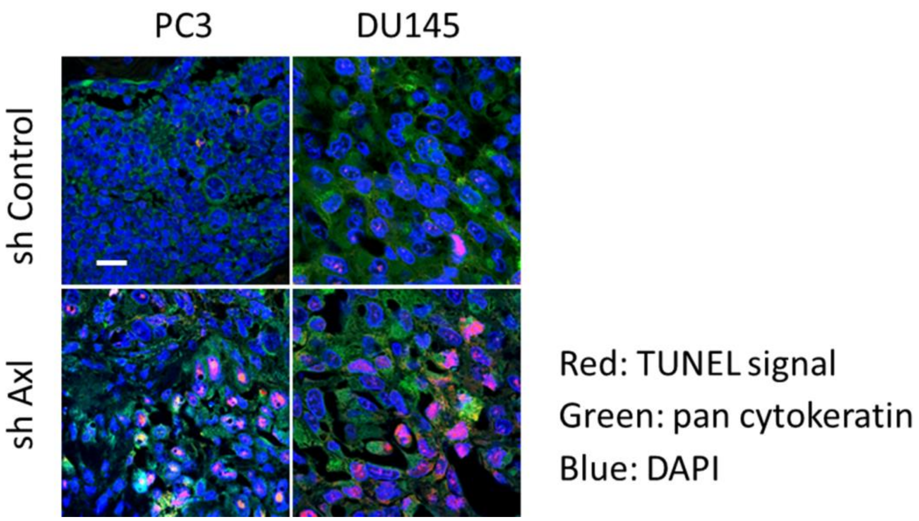

Figure S9: Apoptotic PCa cells in BM defined by TUNEL staining. PCa cells were recognized by pan cytokeratin with green color, and TUNEL signals were shown with red color. Scale bar, 20 μm.
